# Case Report: Unique presentation of iliac vein rupture in an elderly female patient: a multidisciplinary approach to diagnosis and management

**DOI:** 10.3389/fsurg.2026.1774257

**Published:** 2026-03-06

**Authors:** Shixiang Dong, Dongdong Hu, Jing Li, Wen Feng, Weiwei Qian

**Affiliations:** 1 Department of Gynecology, The First People’s Hospital of Lianyungang, Lianyungang, China; 2 Department of Gynecology, The First People’s Hospital of Lianyungang, Affiliated to Kangda College of Nanjing Medical University, Lianyungang, China; 3 Department of Gynecology, Lianyungang Hospital Affiliated to Xuzhou Medical University, Lianyungang, China

**Keywords:** diagnostic challenges, iliac vein rupture, individualized care, multidisciplinary approach, spontaneous rupture

## Abstract

Iliac vein rupture (IVR) is a rare but critical clinical condition often presenting with nonspecific symptoms such as acute abdominal pain and hemorrhagic shock, leading to significant diagnostic challenges. This case report illustrates the complexity of IVR through the clinical course of an elderly female patient with multiple comorbidities, who was ultimately diagnosed with spontaneous IVR following surgical intervention. The case emphasizes the necessity of a multidisciplinary approach involving surgical, vascular, and critical care teams to facilitate timely diagnosis and management. The patient's presentation, characterized by severe pain and hypotension, was initially suggestive of retroperitoneal hemorrhage, yet definitive diagnosis was elusive until surgical exploration was conducted, highlighting the limitations of imaging modalities in certain instances. Furthermore, this case underscores the importance of considering individual risk factors, such as previous pelvic surgeries and underlying venous pathology, in formulating patient-specific care strategies. The successful management of this case not only contributes valuable insights to the existing literature but also advocates for heightened awareness and education among healthcare professionals regarding IVR. Although the rarity of IVR poses challenges to generalizability, it underscores the need for standardized diagnostic protocols and innovative management strategies tailored to high-risk populations. In conclusion, this case serves as a reminder of the critical role of prompt recognition and intervention in improving patient outcomes for those affected by iliac vein rupture.

## Introduction

Iliac vein rupture (IVR) is an infrequent yet life-threatening condition characterized by the acute onset of abdominal pain, hemorrhagic shock, and often vague clinical symptoms that complicate timely diagnosis. While the prevalence of IVR in the general population remains low, it is particularly pertinent to older female patients, who may have underlying risk factors such as decreased venous wall elasticity and previous pelvic surgeries. The occurrence of IVR is notably rare, with only a handful of cases documented in medical literature, emphasizing the critical need for heightened awareness and prompt intervention in suspected cases (1–3).

Patients with IVR typically present with sudden onset of severe pain, hypotension, and signs of shock, which can often escalate to altered mental status and cold extremities (4, 5). The nonspecific nature of these symptoms poses a significant diagnostic challenge, necessitating a high degree of clinical suspicion. Imaging modalities such as contrast-enhanced computed tomography (CT) scans are vital for preoperative diagnosis; however, they may not always yield definitive results, thereby necessitating exploratory surgery for confirmation (6, 7). Furthermore, the presence of pelvic masses or lower limb venous thrombosis can further complicate the clinical picture, leading to a delay in diagnosis and treatment (3).

This case report presents a unique instance of IVR in an elderly female patient, underscoring the complexity and urgency associated with managing such rare conditions. The case highlights the significance of multidisciplinary collaboration in addressing intricate medical scenarios, particularly in high-risk populations. Successful management, as demonstrated here, not only sheds light on the potential for conservative approaches in certain cases but also emphasizes the importance of individualized care strategies that take into account the patient's specific medical history and risk factors (1).

Given the rarity of IVR, this case contributes valuable insights to the existing literature, presenting a compelling argument for the necessity of increased awareness and education surrounding this condition. The successful outcome illustrates the potential for favorable management strategies, even in complex cases, thereby offering guidance for future clinical practice. Ultimately, the knowledge gained from this case may enhance diagnostic acumen and improve patient outcomes in similar scenarios, reinforcing the critical role of thorough assessment and prompt intervention in the management of iliac vein ruptures.

## Case presentation

### Patient information

The patient, a 70-year-old female, was admitted to the emergency department on November 18, 2024, due to a one-day history of pain in the left thigh and experiencing dizziness and a sensation of coldness over the body for five hours. Her medical history includes hypertension and a history of cerebral infarction, and she has been postmenopausal for over 20 years. It should be noted that, although the patient has a history of cerebral infarction, she did not follow medical advice and did not take any anticoagulant medications. On physical examination upon admission, her vital signs were recorded as follows: temperature 36.5 °C, pulse 102 beats per minute, respiratory rate 20 breaths per minute, and blood pressure 62/44 mmHg. The patient appeared lethargic, with cold and moist extremities.

### Clinical findings

**Figure 1:**
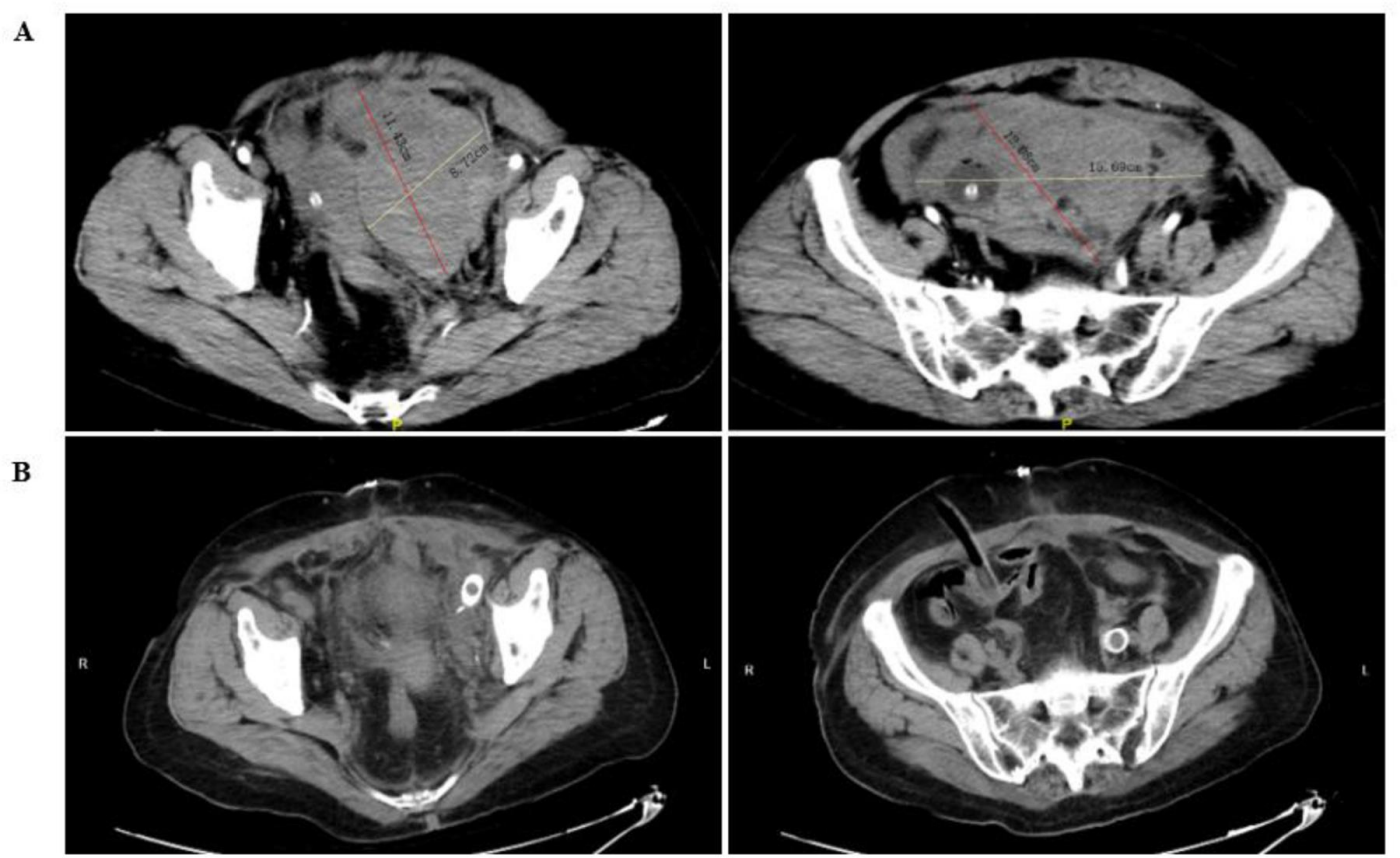
The CT examination image of the patient. **(A)** Shows the image before the surgery, and **(B)** shows the image after the surgery.

Auxiliary examinations revealed a pelvic mass with mixed density, disorganized pelvic structures, multiple pelvic effusions, and perinephric effusion on the left side, as show in Figure 1. Doppler ultrasound of the lower limbs indicated the presence of deep vein thrombosis in the left calf. The blood test upon admission revealed significantly elevated levels of D-dimer (17,903 ng/mL) and fibrin degradation products (187.47 µg/mL), while the hemoglobin level was 68 g/L. No significant abnormalities were detected in other coagulation-related tests. These findings led to a diagnosis of pelvic mass, hypovolemic shock, and lower limb venous thrombosis upon admission.

### Diagnostic assessment

After admission, the patient developed hemorrhagic shock and unstable vital signs, necessitating an emergency laparotomy. Intraoperatively, the uterus and bilateral adnexa appeared normal, and no accumulations of blood were found in the pelvic cavity. However, a large retroperitoneal hematoma, approximately 15 cm in diameter, was identified in the left pelvic region. Upon opening the peritoneum, active bleeding was observed, originating from what appeared to be the left external iliac vein. Gauze was applied to compress the bleeding site as well as the abdominal aorta, and autologous blood transfusion was initiated. During the operation, vascular surgeons noted a rupture of the main trunk of the left external iliac vein with ongoing active bleeding. Given the presence of lower extremity venous thrombosis, they decided to pursue a comprehensive surgical approach, including iliac vein repair (suture), percutaneous lower extremity vein thrombectomy, inferior vena cava filter implantation, and iliac vein stent placement. Postoperatively, the patient was transferred to the ICU for further management, where she experienced multiple episodes of unstable vital signs accompanied by complications such as pulmonary edema and heart failure.

### Therapeutic intervention and outcomes

In the ICU, the patient received symptomatic treatment, including blood transfusions, fluid resuscitation, anti-inflammatory medications, and nutritional support. On the night following the surgery, low molecular weight heparin was immediately administered for anticoagulation, and this treatment was continued until the patient was discharged. After discharge, the patient switched to rivaroxaban tablets for ongoing anticoagulation therapy. During this period, no significant bleeding events occurred. Once her condition stabilized, she was transferred to the gynecology department for continued treatment. Throughout her hospitalization, multiple follow-up examinations were conducted, and treatment plans were adjusted accordingly. The patient gradually resumed her diet and regained physical function, ultimately achieving recovery and being discharged. Regular follow-ups were conducted at 1 month, 3 months, and 6 months after the patient's discharge. To date, the patient has made a good recovery, with no apparent sequelae or complications.

### Insights and lessons learned

Reflecting on this case highlights several critical aspects. The challenges in diagnosing rare conditions such as internal iliac vein rupture, the importance of multidisciplinary collaboration among general surgery, vascular surgery, ICU, anesthesia, transfusion medicine, and gynecology, and the necessity for meticulous perioperative management were all underscored. Additionally, the balance between anticoagulation and bleeding risks in patients with venous thrombosis and the need for individualized therapeutic strategies were emphasized. The case illustrates the significance of optimizing medical resources and decision-making processes, particularly in elderly patients, to enhance recovery outcomes and quality of life. This case serves as a valuable experience for the diagnosis and treatment of similar rare conditions.

## Discussion

The presentation of iliac vein rupture (IVR) often mimics other acute abdominal conditions, leading to diagnostic challenges. In the literature, spontaneous iliac vein rupture is frequently reported without a clear precipitating factor, complicating prompt recognition and treatment. One notable case involved a 62-year-old woman diagnosed with spontaneous iliac vein rupture associated with May-Thurner syndrome, illustrating the variability in clinical presentations and the potential for conservative management (1). In contrast, our case underscores the complexity of IVR in a patient with multiple comorbidities, necessitating a multidisciplinary approach for effective management. Similar to findings in earlier reports, our patient exhibited symptoms consistent with retroperitoneal hemorrhage, yet the diagnosis remained elusive until surgical intervention was performed (3).

Moreover, the management of IVR is particularly nuanced in elderly populations, as demonstrated by a case in which pelvic trauma led to IVR, highlighting the complex interplay between pre-existing conditions and acute events (4). This highlights the importance of a thorough trauma history and preoperative imaging, which proved pivotal in confirming the diagnosis in our patient before surgical intervention (5, 8). The unique presentation of our case relative to others in the literature illustrates both the rarity of such occurrences and the necessity for heightened awareness among clinicians to reduce misdiagnosis and improve outcomes (6).

The diagnosis of iliac vein rupture (IVR) presents significant challenges due to its nonspecific clinical manifestations, which can easily mimic other acute abdominal conditions. A common diagnostic pitfall lies in overlooking IVR in patients with acute pelvic pain, particularly among the elderly who may exhibit atypical presentations arising from comorbidities. For instance, the rarity of spontaneous iliac vein rupture was highlighted in a case study involving an elderly female patient, where the rupture occurred in the absence of a clear precipitating event, ultimately leading to delayed diagnosis and management (2). Additionally, cases of IVR associated with predisposing factors such as venous hypertension further complicate the clinical picture, necessitating enhanced awareness among clinicians to consider IVR in differential diagnoses of acute abdominal complaints (3, 5). The importance of comprehensive history-taking, including inquiries into previous thrombotic events and trauma, is underscored in literature, which emphasizes the critical role of meticulous preoperative imaging in confirming the diagnosis before surgical intervention (1).

Furthermore, the management of IVR necessitates a multidisciplinary approach due to the complexity of cases, particularly in older patients who often present with multiple comorbidities. A coordinated effort among surgical, vascular, and critical care teams can significantly enhance patient outcomes, as demonstrated in instances where collaborative management strategies were employed successfully (6). The optimization of perioperative care is particularly crucial, focusing on fluid management, coagulation status, and organ function support to mitigate postoperative complications, which are prevalent in the elderly population (8, 9). The interplay between anticoagulation therapy and the risk of postoperative bleeding further necessitates individualized management plans, as indicated in previous studies (10, 11). These insights into diagnostic and management strategies highlight the importance of heightened clinical vigilance and a collaborative healthcare approach in addressing the complexities associated with IVR.

In summary, the presented case of iliac vein rupture (IVR) not only underscores the intricate nature of diagnosing and managing this rare condition but also reflects the critical importance of a multidisciplinary approach in patient care. The key decisions made throughout the diagnostic and therapeutic processes were influenced by the patient's complex medical history and presenting symptoms. The successful outcome was primarily attributed to the collaborative efforts of surgical, vascular, and critical care teams, emphasizing the necessity of tailored interventions that consider individual risk factors and comorbidities. This case serves as a poignant reminder that timely recognition and intervention in IVR cases can significantly improve patient prognosis, thereby reinforcing the need for ongoing education and awareness among healthcare professionals regarding this elusive condition.

Despite the valuable insights gained from this case, several limitations warrant consideration. The rarity of IVR poses inherent challenges to generalizability, as each case may present with unique clinical features and complexities. Additionally, the reliance on surgical intervention for definitive diagnosis in our case may not always be feasible in other clinical settings. Future research should aim to establish standardized diagnostic protocols and explore innovative management strategies that can enhance the identification and treatment of IVR, particularly in high-risk populations. Ultimately, a commitment to advancing our understanding of IVR will be crucial in refining clinical practices and improving outcomes for affected patients.

## Data Availability

The original contributions presented in the study are included in the article/Supplementary Material, further inquiries can be directed to the corresponding author.
